# The impact of underdiagnosis and delay in care for patients with moderate and severe aortic stenosis: A real-world database cohort study

**DOI:** 10.1016/j.ahjo.2026.100884

**Published:** 2026-09-08

**Authors:** Ben Peterson, Jordan B. Strom, Michael Ryan, Candace Gunnarsson, Soumya G. Chikermane, Seth Clancy, Sammy Elmariah

**Affiliations:** a Intermountain Medical Center, Salt Lake City, UT, United States of America; b Richard A. and Susan F. Smith Center for Outcomes Research in Cardiology, Division of Cardiovascular Medicine, Beth Israel Deaconess Medical Center, Harvard Medical School, United States of America; c MPR Consulting, Cincinnati, OH, United States of America; d Gunnarsson Consulting, Boise, ID, United States of America; e Edwards Lifesciences, Irvine, CA, United States of America; f UCSF Cardiac Catheterization Laboratory University of California, San Francisco, San Francisco, CA, United States of America

**Keywords:** Aortic stenosis, Echocardiography, Underdiagnosed AS, Diagnostic disparities, Delayed care

## Abstract

**Background:**

Despite advances in diagnostic capabilities and therapeutic options for aortic stenosis (AS), evidence suggests substantial underdiagnosis and care delays in real-world practice. This study examines patterns and disparities in AS diagnosis following echocardiographic identification, quantifying delays in diagnosis and subsequent care.

**Methods:**

Using the Optum® Market Clarity Dataset (2016–2023), we identified patients with first echocardiographic evidence of moderate or severe AS. Time-to-event analyses examined delays between echocardiographic findings and formal diagnosis (defined by ICD code assignment), subsequent imaging, and interventions. Analyses were stratified by severity, demographics, and care setting.

**Results:**

Among 2366 moderate and 1327 severe AS patients, 22.0% and 38.3% received formal diagnoses within one year of echocardiogram, respectively. Cumulative incidence of AS diagnosis at one year was lower in women vs men (moderate: 17.3% vs 27.8%; severe: 33.8% vs 43.0%) and non-White vs White patients (moderate: 11.4% vs 24.6%; severe: 25.8% vs 40.4%; all *P* ≤ 0.0001). Formal diagnosis was associated with increased likelihood of subsequent care (composite of follow-up imaging or aortic valve replacement) (moderate AS: HR 1.68, *P* < 0.0001; severe AS: HR 2.25, *P* < 0.0001).

**Conclusions:**

This analysis reveals substantial delays in formal ICD-coded AS diagnosis following its echocardiographic detection, with concerning disparities across demographic groups. These findings suggest gaps between clinical recognition and formal documentation of AS, with implications for care continuum.

**Clinical perspectives:**

What Is New?

• Using a large, real-world integrated claims and EMR database, this study demonstrates that fewer than one-quarter of patients with echocardiographically confirmed moderate AS and fewer than 40% with severe AS receive a formal ICD-coded diagnosis within one year, with substantially lower rates among women and non-White patients.

What Are the Clinical Implications?

• These findings reveal a critical gap between echocardiographic detection of AS and formal clinical documentation, suggesting that administrative claims data alone may substantially underestimate the true burden of AS in the population.
• Formal AS diagnosis was independently associated with a significantly higher likelihood of receiving subsequent care — including surveillance imaging and aortic valve replacement — underscoring that closing the documentation gap may be a meaningful lever for improving outcomes and reducing disparities in AS management.

## Non-standard Abbreviations and Acronyms

AS: aortic stenosis
AVA: aortic valve area
AVR: aortic valve replacement
CPT: Current Procedural Terminology
ECI: Elixhauser Comorbidity Index
EMR: electronic medical records
EPN: electronic provider notifications
ICD: international classification of diseases
TEE: transesophageal
TTE: transthoracic

## 1 Introduction

Aortic stenosis (AS) represents a significant cardiovascular health burden, affecting approximately 2%–7% of adults over 65 years of age in developed nations [1]. The condition is characterized by progressive narrowing of the aortic valve, leading to increased morbidity and mortality when left untreated. Indeed, Généreux et al. demonstrated that untreated AS is associated with substantial mortality risk, with four-year all-cause mortality rates of 25.0%, 33.5%, and 44.9% for mild, moderate, and severe AS, respectively [1]. These rates are even higher in symptomatic severe AS patients, with the landmark PARTNER 1 trial demonstrating approximately 50% mortality at one year for medically managed symptomatic severe AS patients [2], [3], [4].

Despite effective treatment options, AS remains underdiagnosed in clinical practice [5], [6], [7]. Crousillat et al. found that only 37.4% of patients with echocardiographically confirmed AS received a formal international classification of diseases (ICD) diagnosis within one year, with even lower rates among racial and ethnic minorities [5].

Given growing evidence of disparities in cardiovascular care by sex, race, and age [5], [8], we evaluated temporal patterns and disparities in AS diagnosis following echocardiographic identification of moderate or severe AS and assessed the relationship between formal diagnosis and follow-up care.

## 2 Methods

### 2.1 Data source

This study used Optum® Market Clarity Database (claims + electronic medical records [EMR]) from January 1, 2016, to December 31, 2023. The dataset is a robust, clinically rich, and payer-complete longitudinal integrated dataset that deterministically links medical and pharmacy claims with electronic health records. The dataset incorporates natural language processing applied to unstructured clinical notes across 100 million patient encounters. Variables include but are not limited to in-office assessments and measurements, diagnosis codes, signs and symptoms, biomarkers, labs, and test results. All data used to perform this analysis were de-identified and accessed in compliance with the Health Insurance Portability and Accountability Act. As a retrospective analysis of a de-identified database, the research was exempt from IRB review under 45 CFR 46.101(b)(4).

### 2.2 Study cohort and patient population

The study population consisted of two mutually exclusive cohorts (severe and moderate AS) derived from structured fields based on a patient's initial echocardiographic parameters within the electronic health records component of the Market Clarity Database.

Patients were required to have a record of an echocardiogram (inclusive of both transthoracic [TTE] and transesophageal [TEE] procedures) via ICD-10 procedure or Current Procedural Terminology (CPT) coding in the Market Clarity database from 2016 to 2023. First record of any echocardiogram was considered time zero. Patients were included if 18 years of age or older with at least 12 months of continuous health plan enrollment (to establish baseline comorbidities) prior to time zero and with echocardiogram demonstrating moderate or severe AS echocardiography. Severity of AS was defined as: moderate AS = peak velocity between 3 and 4 m/s, or a mean gradient between 20 and 40 mmHg, or aortic valve area (AVA) between 1 and 1.5 cm^2^. Severe AS = peak velocity ≥ 4 m/s, or mean gradient ≥40 mmHg, or AVA ≤1 cm^2^. For discordant measurements, classification was based on the more severe parameter [9].

Patients were excluded if: (1) they had a record of an AS diagnosis or record of aortic valve replacement (AVR) (either via claims or in EMR) prior to time zero; (2) they had a record of hospice care or being in a skilled nursing facility during the baseline period or time zero.

### 2.3 Endpoints

The primary endpoint was formal AS diagnosis as defined by assignment of ICD billing code for aortic valve disease within one year of the index echocardiogram (see Appendix A for all ICD10 and CPT coding detail). Secondary endpoints included time to surveillance echocardiogram, time to AVR, and time to the composite of surveillance echocardiogram or AVR. All secondary endpoints were evaluated over the entire follow-up period using Cox proportional hazards models.

### 2.4 Variables of interest

Baseline characteristics included age at index echocardiogram, sex (male/female), race (categorized as Asian, Black, Caucasian, or unknown), ethnicity (Hispanic, non-Hispanic, other/unknown) and geographic region (Northeast, Midwest, South, West, or unknown). The clinical setting of the index echocardiogram was also included, categorized as either inpatient or outpatient.

Comorbid conditions were assessed at baseline using the Elixhauser Comorbidity Index (ECI), a comprehensive measure that encompasses 31 distinct categories of comorbidities associated with mortality [10]. These conditions were identified through diagnosis codes present at baseline and included: congestive heart failure, cardiac arrhythmia, valvular disease, pulmonary circulation disorders, peripheral vascular disorders, hypertension (both uncomplicated and complicated), chronic pulmonary disease, diabetes (uncomplicated and complicated), renal failure, liver disease, obesity, fluid and electrolyte disorders, blood loss anemia, deficiency anemia, hypothyroidism, depression, psychoses, and other neurological disorders. Additional comorbidities at baseline included AIDS/HIV, lymphoma, metastatic cancer, solid tumors without metastasis, rheumatoid arthritis, coagulopathy, paralysis, weight loss, alcohol abuse, drug abuse, and peptic ulcer disease (excluding bleeding). A composite ECI score was calculated based on these conditions to provide an overall measure of comorbidity burden.

### 2.5 Statistical analysis

Baseline characteristics, including demographics and comorbid conditions, were summarized for the moderate and severe AS cohorts, stratified by subsequent receipt of formal AS diagnosis in claims data. Descriptive statistics included means and standard deviations for continuous variables and frequencies and proportions for categorical variables.

Time-to-event analyses were conducted using Kaplan-Meier curves to estimate the probability of receiving an AS diagnosis (primary outcome) within one year from the index echocardiogram showing either moderate or severe AS. These unadjusted curves were generated for the overall population; we then examined potential disparities in care patterns by stratifying the overall population by key subgroups, including site of care (inpatient vs outpatient), sex (male vs female), race (White vs non-White), ethnicity (Hispanic vs non-Hispanic), and age (<75 vs ≥75 years, and <65 vs 65–80 vs >80 years). Log-rank tests were performed to assess statistical significance of differences between subgroups.

Next, secondary outcomes (time to second echocardiogram, time to AVR, and time to either second echocardiogram or AVR) were modeled using multivariable Cox proportional hazards models, where end of data and death are considered censoring events. The main explanatory variable of interest was the time varying covariate, time to AS diagnosis. The models were adjusted for patient demographics and comorbidity burden using the ECI.

## 3 Results

**Fig. 1:**
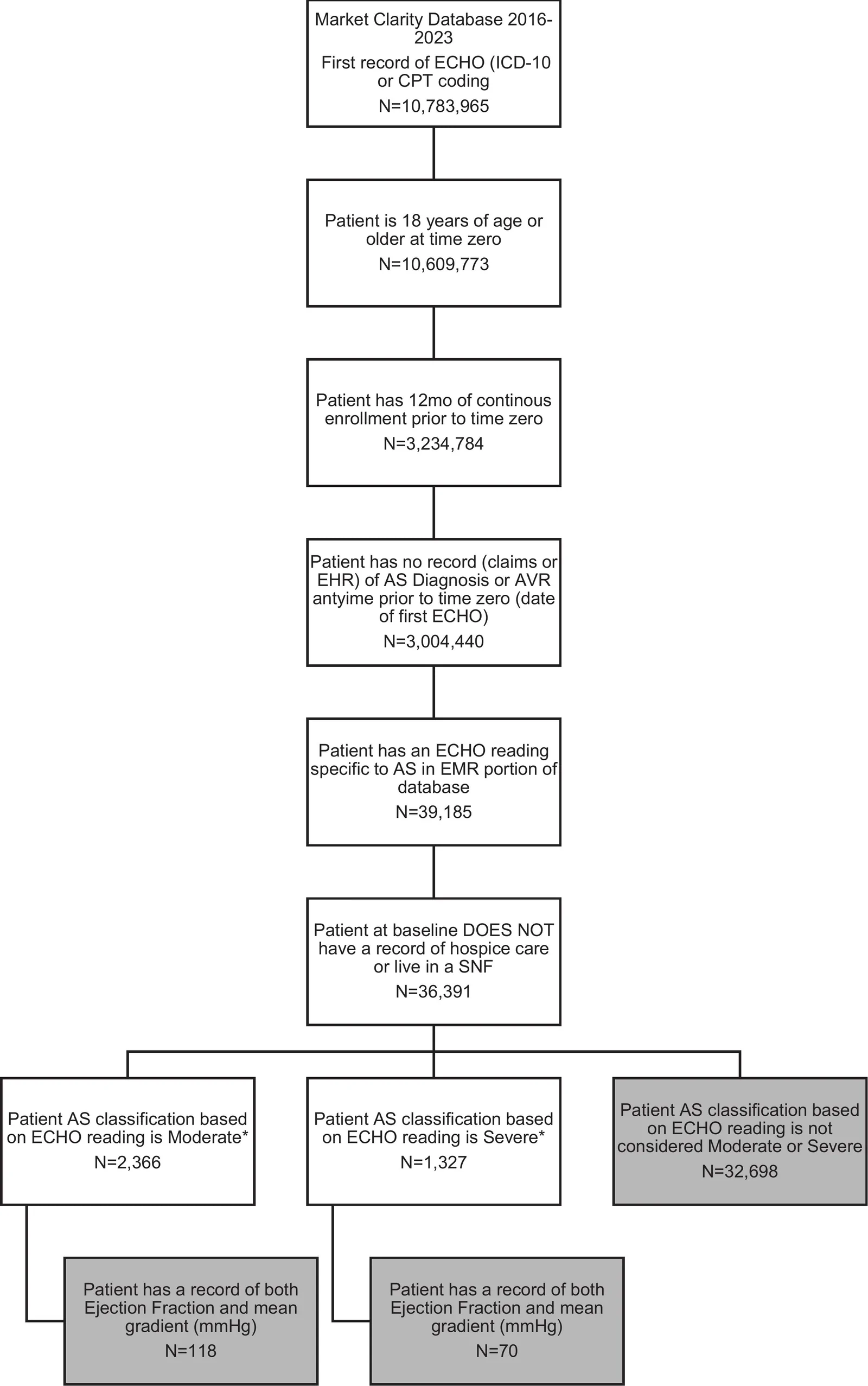
Study attrition diagram. ECHO = echocardiogram; ESRD = end stage renal disease; SNIF = skilled nursing facility; Moderate AS = peak velocity between 3 and 4 m/s, mean gradient between 20 and 40 mmHg, or AVA between 1 and 1.5 cm^2^; Severe AS = peak velocity ≥ 4 m/s, mean gradient ≥40 mmHg, or AVA ≤1 cm^2^.

The study population comprised 2366 moderate AS and 1327 severe AS patients based on echocardiographic findings (Fig. 1).

Table 1 presents baseline characteristics for both cohorts stratified by receipt of formal AS diagnosis. The moderate AS cohort (*n* = 2366) had a mean age of 68.3 years and was 56% female and 79% White. The severe AS cohort (*n* = 1327) had a mean age of 69.6 years and was 53% female and 85% White. Mean ECI scores were 4.0 for moderate AS and 3.6 for severe AS patients. Patients receiving AS diagnoses were older in both severity groups (moderate: 72.0 vs 67.1 years [*P* < 0.0001]; severe: 72.2 vs 67.6 years [*P* < 0.0001]), and males were overrepresented among diagnosed cases (moderate: 53.3% vs 40.2% [*P* < 0.0001]; severe: 52.8% vs 42.3% [*P* = 0.0002]). The racial disparity observed in time-to-diagnosis curves was reflected in the higher proportion of White patients among diagnosed cases (moderate: 89.0% vs 75.7% [*P* < 0.0001]; severe: 89.5% vs 80.3% [*P* < 0.0001]). Notably, the ECI scores were similar between diagnosed and undiagnosed groups (range: 3.29–4.03).

**Table 1:** Patient characteristics with ECHO findings of moderate and severe AS stratified by those that receive an AS diagnosis in claims versus those that do not.

	Moderate AS			Severe AS		
	Dx of AS anytime post	No Dx of AS Anytime post	*P*-value	Dx of AS anytime post	No Dx of AS anytime post	P-value
Total patients	664	1702		618	709	
Age mean (SD)	71.98 (10.94)	67.14 (13.89)	<0.0001	72.19 (11.48)	67.64 (14.16)	<0.0001
Gender						
Female	310 (46.69%)	1018 (59.81%)	<0.0001	292 (47.25%)	409 (57.69%)	0.0002
Male	354 (53.31%)	684 (40.19%)		326 (52.75%)	300 (42.31%)	
Race						
Asian	4 (0.60%)	18 (1.06%)	<0.0001	0 (0.00%)	11 (1.55%)	<0.0001
Black	54 (8.13%)	331 (19.45%)		43 (6.96%)	109 (15.37%)	
Caucasian	591 (89.01%)	1288 (75.68%)		553 (89.48%)	569 (80.25%)	
Other/unknown	15 (2.26%)	65 (3.82%)		22 (3.56%)	20 (2.82%)	
Ethnicity						
Hispanic	11 (1.66%)	39 (2.29%)	0.0917	17 (2.75%)	17 (2.40%)	0.0060
Non-Hispanic	605 (91.11%)	1576 (92.60%)		542 (87.70%)	656 (92.52%)	
Other/unknown	48 (7.23%)	87 (5.11%)		59 (9.55%)	36 (5.08%)	
Region						
Midwest	240 (36.14%)	423 (24.85%)	<0.0001	311 (50.32%)	249 (35.12%)	<0.0001
Northeast	64 (9.64%)	134 (7.87%)		90 (14.56%)	81 (11.42%)	
South	257 (38.70%)	934 (54.88%)		144 (23.30%)	313 (44.15%)	
West	89 (13.40%)	176 (10.34%)		56 (9.06%)	49 (6.91%)	
Unknown	14 (2.11%)	35 (2.06%)		17 (2.75%)	17 (2.40%)	
Payor						
Commercial	206 (31.02%)	608 (35.72%)	0.0101	208 (33.66%)	267 (37.66%)	0.0235
Medicare	425 (64.01%)	974 (57.23%)		361 (58.41%)	390 (55.01%)	
Medicaid	29 (4.37%)	112 (6.58%)		43 (6.96%)	52 (7.33%)	
Other/unknown	4 (0.60%)	8 (0.47%)		6 (0.97%)	0 (0.00%)	
Elixhauser severity score mean (SD)	4.02 (3.03)	4.03 (3.06)	0.9232	3.29 (2.68)	3.76 (2.92)	0.0026

**Fig. 2:**
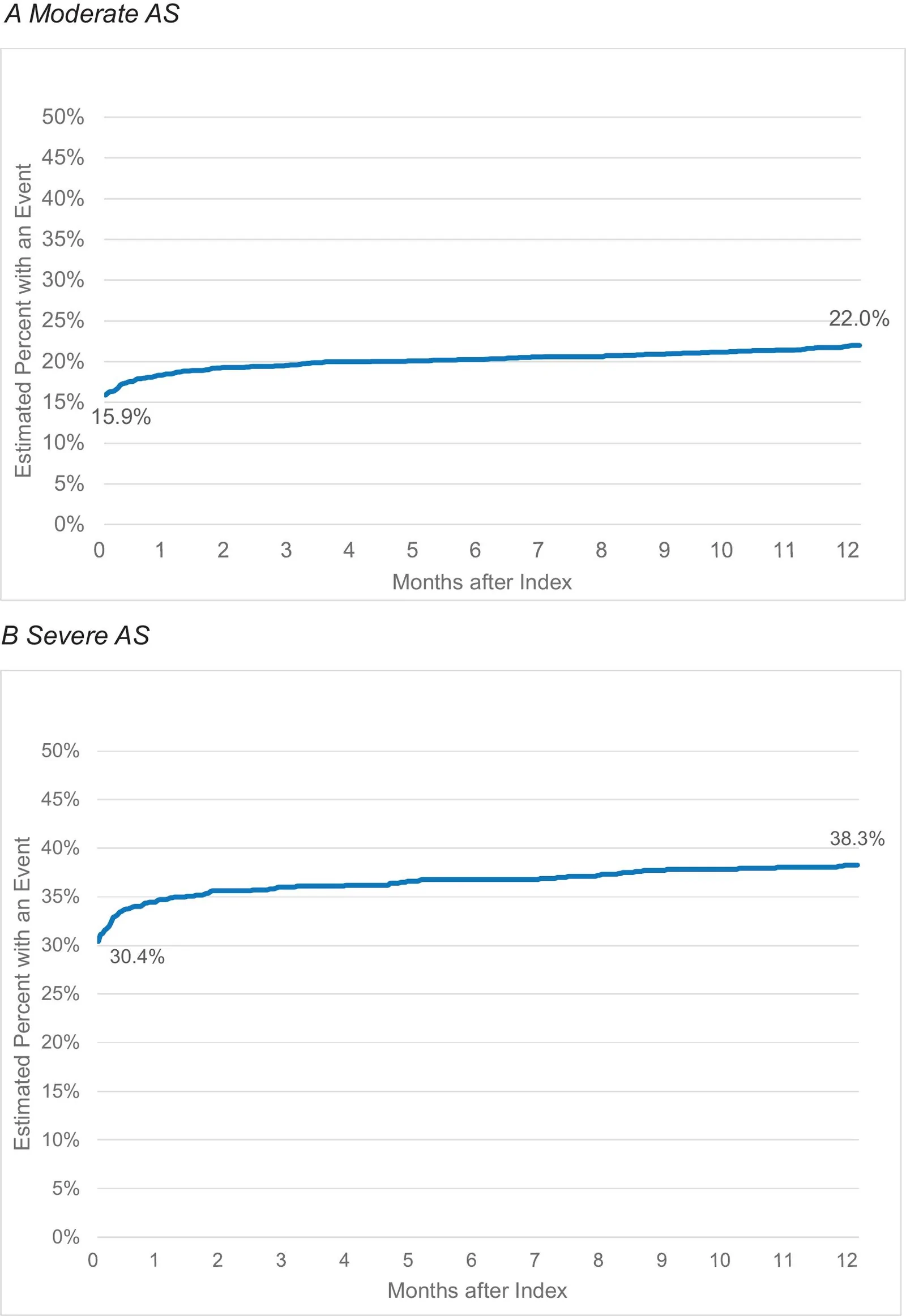
A-B: Time to an AS diagnosis from ECHO finding of moderate and severe at 1 year.

**Fig. 3:**
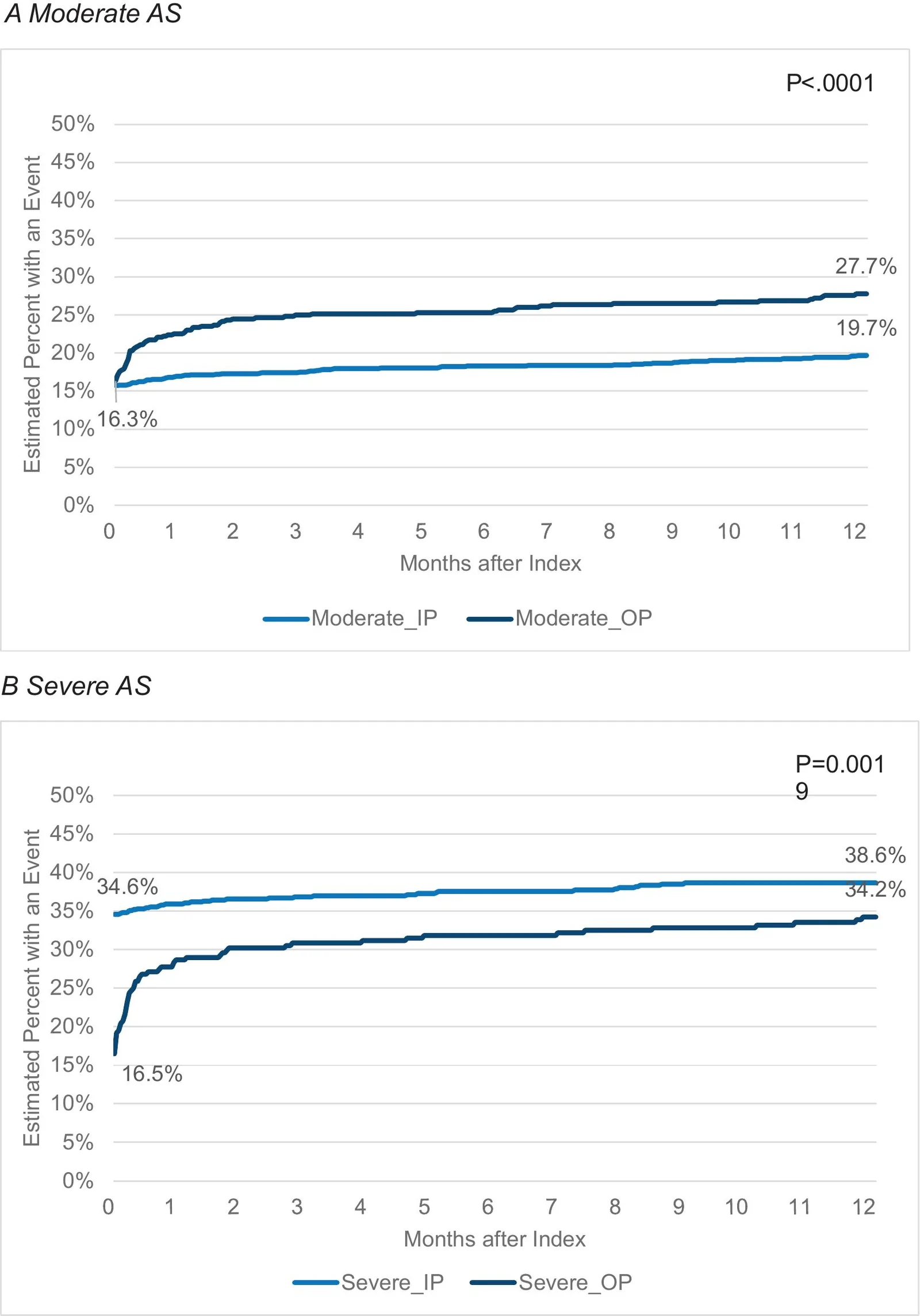
A-B: Time to an AS diagnosis from ECHO finding of moderate and severe at 1 year by site of care (ECHO done inpatient versus outpatient).

At one year following initial echocardiographic findings, 22.0% of patients with moderate AS and 38.3% of patients with severe AS received a formal diagnosis in claims data. A majority of severe or moderate AS patients who received a formal diagnosis of AS received it within the first 30 days (34.7% and 18.5% respectively; Fig. 2A and B). In moderate AS, diagnosis rates at one year were higher in outpatient than inpatient settings (27.7% vs 19.7% [*P* < 0.0001]; Fig. 3A). Conversely, in severe AS, inpatient diagnosis rates were higher than outpatient rates (38.6% vs 34.2% [*P* = 0.0019]; Fig. 3B) at one year.

**Fig. 4:**
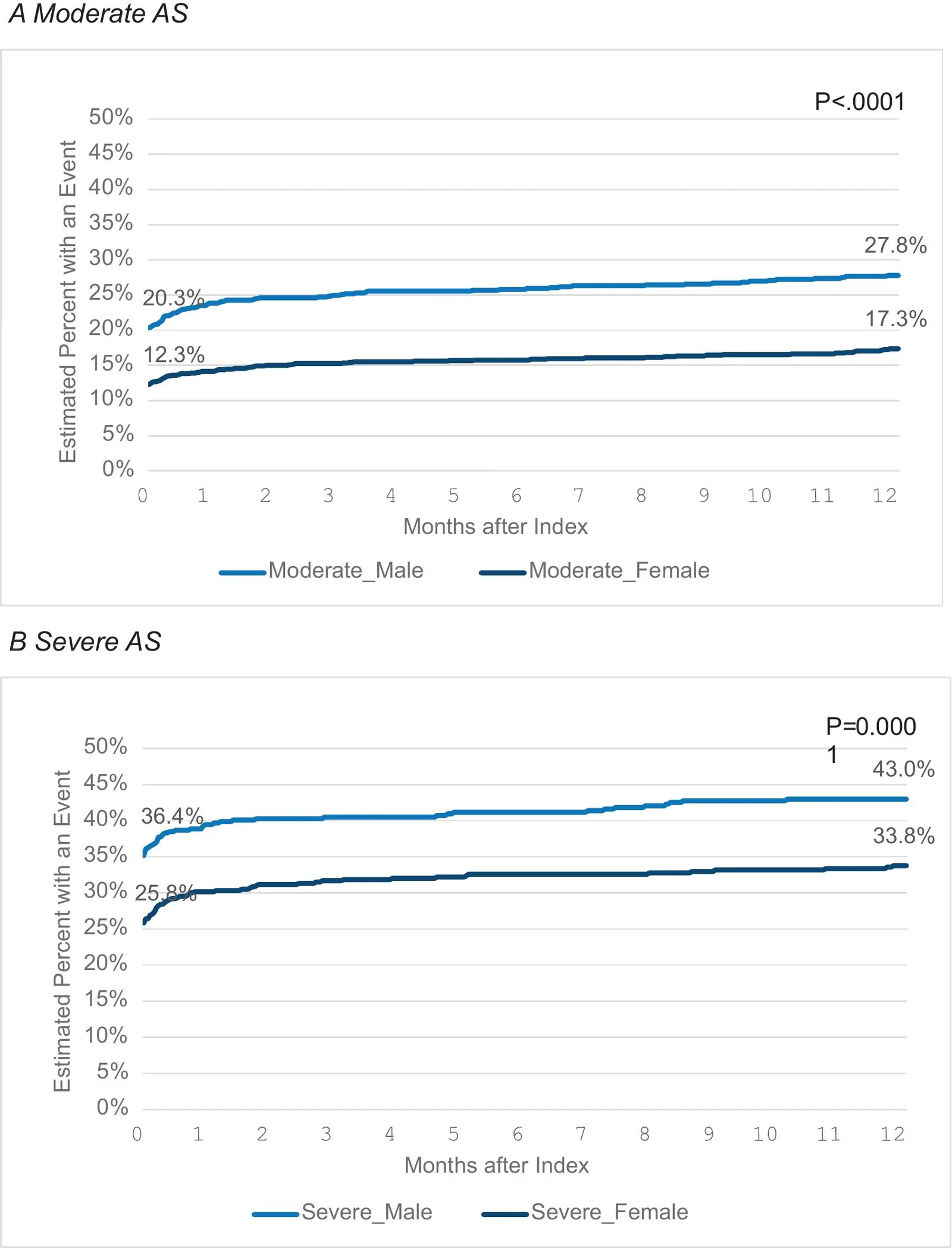
A-B: Time to an AS diagnosis from ECHO finding of moderate and severe at 1 year by sex (male versus female).

In moderate AS, men were significantly more likely to receive a diagnosis compared to women (27.8% vs 17.3% [*P* < 0.0001]; Fig. 4A) at one year, and this pattern persisted in severe AS (43.0% vs 33.8% [*P* = 0.0001]; Fig. 4B).

**Fig. 5:**
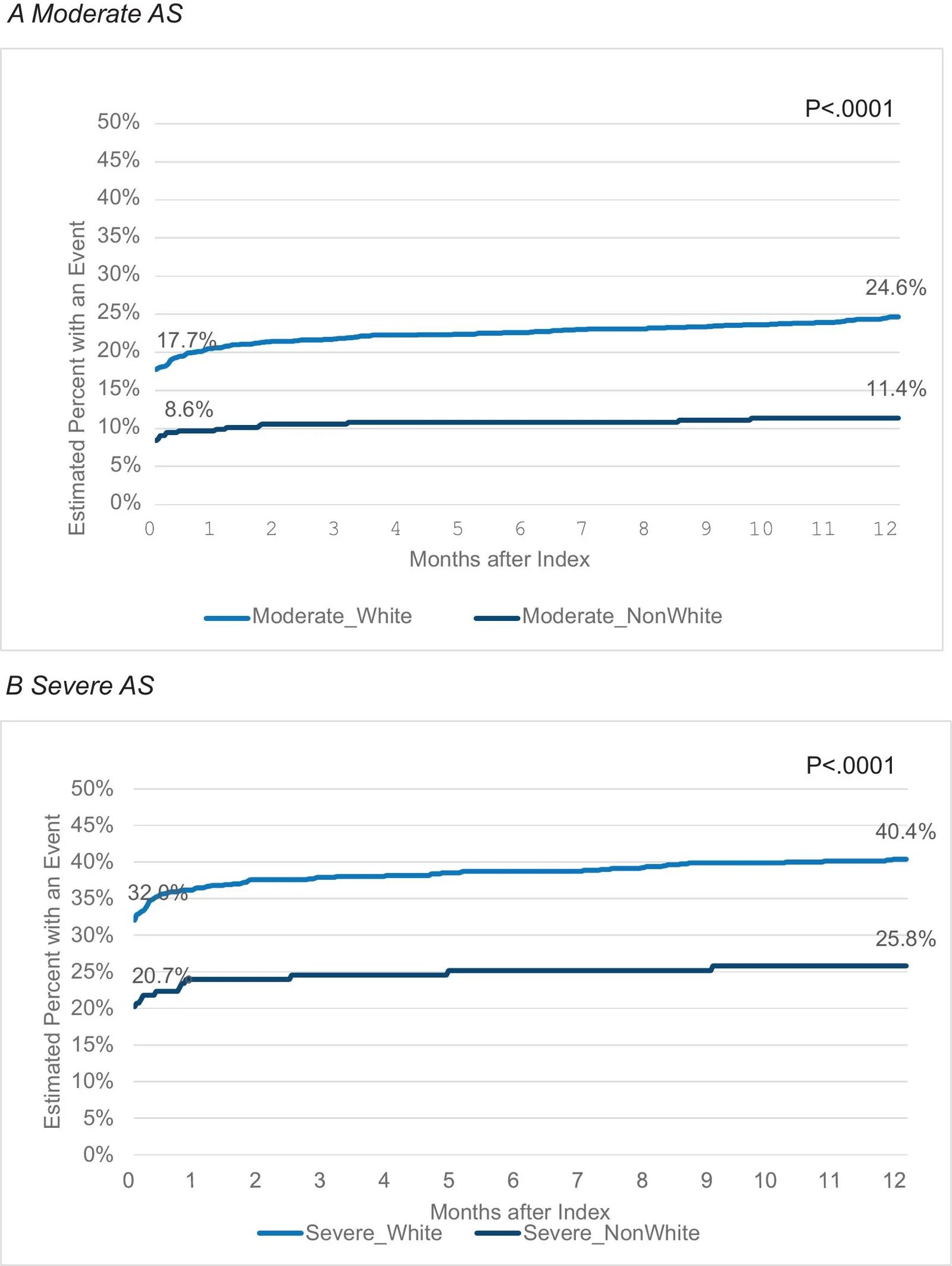

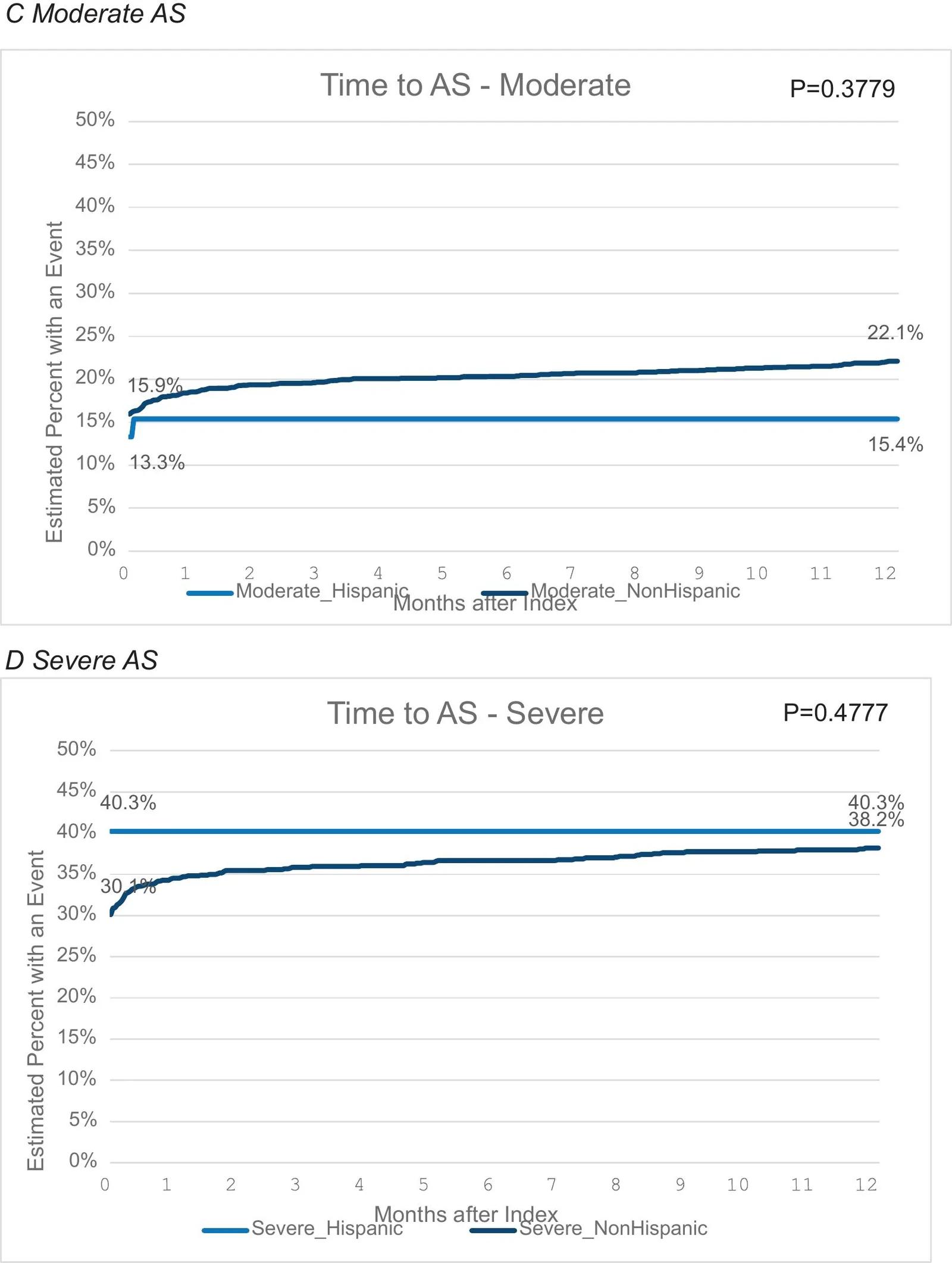
A-B: Time to an AS diagnosis from ECHO finding of moderate and severe at 1 year by race (White versus non-White). C-D: Time to an AS diagnosis from ECHO finding of moderate and severe at 1 year by ethnicity (Hispanic versus non-Hispanic).

White patients had markedly higher diagnosis rates compared to non-White patients in both moderate (24.6% vs 11.4% [*P* < 0.0001]; Fig. 5A) and severe AS (40.4% vs 25.8% [*P* < 0.0001]; Fig. 5B) at one year. Similarly, for ethnic comparisons, non-Hispanic patients showed higher diagnosis rates compared to Hispanic patients in both moderate and severe AS groups (Fig. 5C and D).

**Fig. 6:**
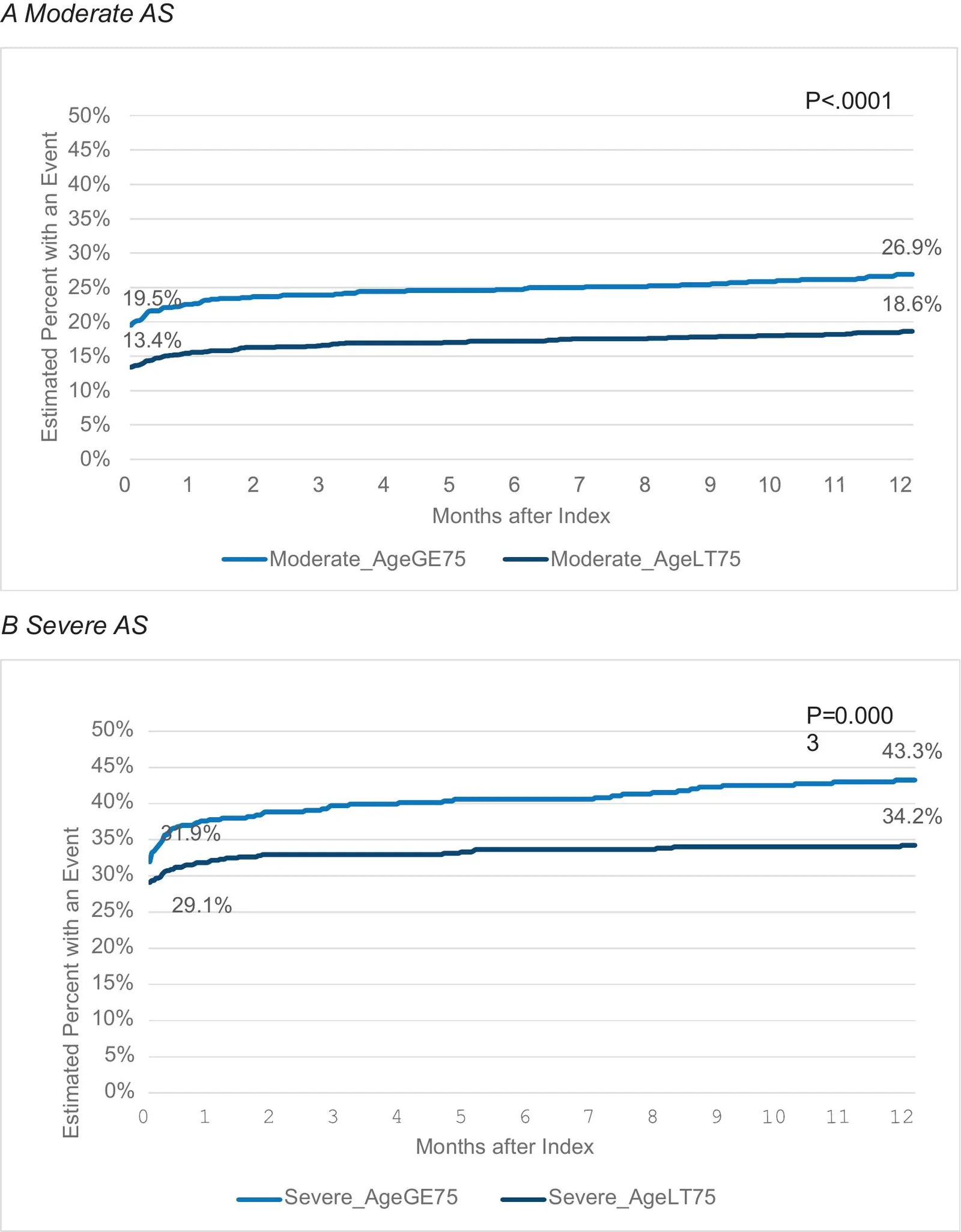

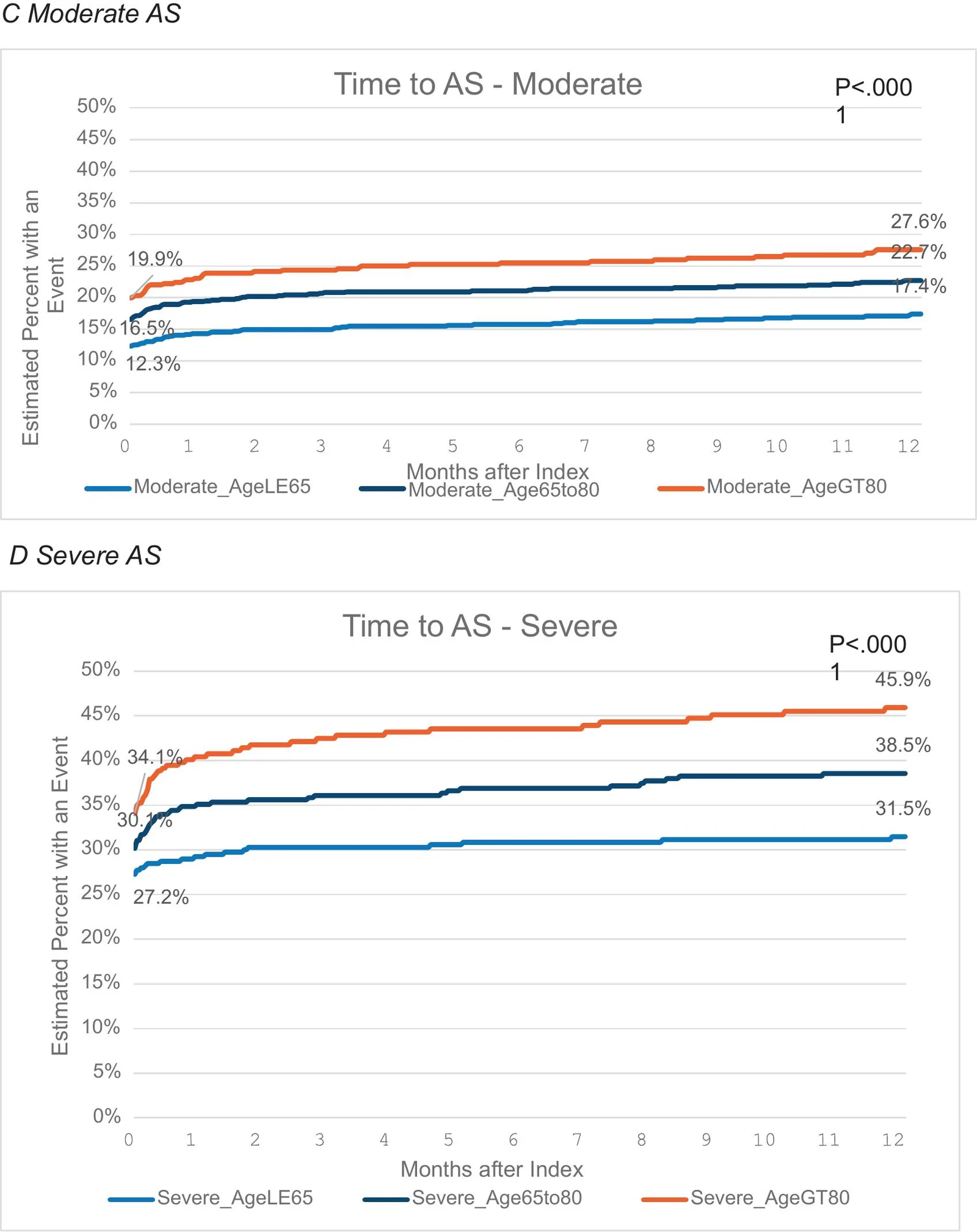
A-B: Time to an AS diagnosis from ECHO finding of moderate and severe at 1 year by patients 75 years of age and older versus less than 75. C-D: Time to an AS diagnosis from ECHO finding of moderate and severe at 1 year by patients aged less than 65, 65 to 80, and greater than 80.

Older patients (≥75 years) showed higher diagnosis rates compared to younger patients (<75 years) in both moderate (26.9% vs 18.6% [*P* < 0.0001]; Fig. 6A) and severe AS (43.3% vs 34.2% [*P* = 0.0003]; Fig. 6B) at one year. When broken down further into three age groups (<65, 65–80, >80), the highest diagnosis rates were consistently seen in the oldest age group (>80), followed by the 65–80 group, with patients under 65 showing the lowest rates of diagnosis in both moderate and severe AS (Fig. 6C and D).

The median time to surveillance echocardiogram showed notable disparities across patient subgroups. Overall, severe AS patients received follow-up echocardiogram earlier than moderate AS (385 vs 522 days [*P* < 0.0001]; Table 2A). Significant gender disparities were observed, with women experiencing longer delays to follow-up compared to men in both moderate (662 vs 396 days [*P* < 0.0001]) and severe AS (494 vs 290 days [*P* < 0.0001]). Patients found to have severe AS in the inpatient setting underwent surveillance echocardiogram earlier than those with outpatient echocardiogram (343 vs 490 days [*P* = 0.0135]). Similar patterns were observed for the composite secondary endpoint of surveillance echocardiogram or AVR (see Table 2A, Table 2B).

**Table 2A:** Summary findings for Median Days to 2nd ECHO overall and by sub-populations of interest.

	Moderate AS	Severe AS	P-value
	Based on ECHO findings	Based on ECHO findings	
	Overall	Overall	
Total patient count	2366	1327	
Median Days to 2nd ECHO overall	522	385	<0.0001
Median Days to 2nd ECHO women	662	494	0.0046
Median Days to 2nd ECHO men	396	290	0.0021
Median Days to 2nd ECHO white	553	377	<0.0001
Median Days to 2nd ECHO non-white	406	420	0.5280
Median Days to 2nd ECHO Hispanic	380	320	0.5828
Median Days to 2nd ECHO non-Hispanic	526	387	<0.0001
Median Days to 2nd ECHO <75 years	486	358	0.0009
Median Days to 2nd ECHO ≥75 years	607	418	0.0013
Median Days to 2nd ECHO <65 years	469	342	0.0069
Median Days to 2nd ECHO 65–80 years	528	359	0.0007
Median Days to 2nd ECHO >80 years	634	514	0.0550
Median Days to 2nd ECHO or AVR overall	528	392	0.0001
Median Days to 2nd ECHO or AVR women	667	505	0.0100
Median Days to 2nd ECHO or AVR men	403	297	0.0038
Median Days to 2nd ECHO or AVR white	560	386	<0.0001
Median Days to 2nd ECHO or AVR non-white	416	442	0.6835
Median Days to 2nd ECHO or AVR Hispanic	380	320	0.5342
Median Days to 2nd ECHO or AVR non-Hispanic	530	394	0.0001
Median Days to 2nd ECHO or AVR <75 years	495	370	0.0036
Median Days to 2nd ECHO or AVR ≥75 years	607	425	0.0024
Median Days to 2nd ECHO or AVR <65 years	478	370	0.0287
Median Days to 2nd ECHO or AVR 65–80 years	531	365	0.0008
Median Days to 2nd ECHO or AVR >80 years	634	516	0.0883

**Table 2B:** Summary findings for Median Days to 2nd ECHO by sub-populations of interest, inpatient vs outpatient.

	Moderate AS			Severe AS		
	Based on ECHO findings			Based on ECHO findings		
	ECHO inpatient	ECHO outpatient	P-value	ECHO inpatient	ECHO outpatient	P-value
Total patient count	1725	641		992	335	
Median Days to 2nd ECHO overall	503	563	0.2443	343	490	0.0135
Median Days to 2nd ECHO women	617	726	0.1697	465	565	0.6514
Median Days to 2nd ECHO men	399	393	0.8719	239	414	0.0103
Median Days to 2nd ECHO white	530	600	0.2558	341	459	0.0435
Median Days to 2nd ECHO non-white	406	421	0.8785	365	846	0.1006
Median Days to 2nd ECHO Hispanic	380	373	0.7277	298	–	0.2023
Median Days to 2nd ECHO non-Hispanic	506	563	0.2709	347	490	0.0224
Median Days to 2nd ECHO <75 years	447	563	0.1965	314	477	0.0178
Median Days to 2nd ECHO ≥75 years	614	565	0.7464	393	490	0.3282
Median Days to 2nd ECHO <65 years	436	657	0.1602	298	505	0.0516
Median Days to 2nd ECHO 65–80 years	494	600	0.1595	302	534	0.0061
Median Days to 2nd ECHO >80 years	706	455	0.1713	516	455	0.4458
Median Days to 2nd ECHO or AVR overall	507	563	0.3298	359	490	0.0344
Median Days to 2nd ECHO or AVR women	617	726	0.1865	494	565	0.8252
Median Days to 2nd ECHO or AVR men	414	393	0.9724	259	414	0.0144
Median Days to 2nd ECHO or AVR white	545	600	0.3487	352	459	0.0878
Median Days to 2nd ECHO or AVR non-white	406	421	0.8467	391	846	0.1361
Median Days to 2nd ECHO or AVR Hispanic	416	373	0.803	298	–	0.1911
Median Days to 2nd ECHO or AVR non-Hispanic	511	563	0.3556	366	490	0.0580
Median Days to 2nd ECHO or AVR <75 years	462	563	0.2889	335	477	0.0460
Median Days to 2nd ECHO or AVR ≥75 years	614	565	0.7583	396	490	0.3806
Median Days to 2nd ECHO or AVR <65 years	442	657	0.2314	335	505	0.1337
Median Days to 2nd ECHO or AVR 65–80 years	496	600	0.1894	311	534	0.0065
Median Days to 2nd ECHO or AVR >80 years	706	455	0.1713	530	455	0.3493

In Cox proportional hazards models with AS as a time varying covariate (see Table 3) for the secondary outcomes, receipt of a formal AS diagnosis was strongly associated with subsequent care patterns. Moderate AS patients who received a diagnosis were 68% more likely to undergo a second surveillance echocardiogram (HR: 1.68, 95% CI: 1.50–1.89, *P* < 0.0001), while diagnosed severe AS patients showed a 125% increased likelihood (HR: 2.25, 95% CI: 1.95–2.60 [*P* < 0.0001]). Receipt of an AS diagnosis associated with increased likelihood of undergoing subsequent AVR for moderate (HR 102.30; 95% CI: 37.12–281.70) and severe AS (HR 219.50; 95% CI: 54.41–885.90). Rates of subsequent care differed markedly by diagnosis status. Among moderate AS patients, a second echocardiogram occurred in 77.4% of diagnosed versus 56.3% of undiagnosed patients, and AVR in 18.7% versus 0.2%, respectively. Among severe AS patients, a second echocardiogram occurred in 74.3% of diagnosed versus 55.0% of undiagnosed patients, and AVR in 40.6% versus 0.3%.

**Table 3:** Multivariable model results for secondary outcomes of interest (time to 2nd ECHO, 2nd ECHO or AVR) with AS diagnosis as a time varying covariate.

	Outcome	Hazard ratio	*P* value
Moderate AS based on ECHO	ECHO2nd	1.68 (1.50–1.89)	<0.0001
	AVR	102.30 (37.12–281.70)	<0.0001
	AVR OR ECHO2ND	1.67 (1.48–1.87)	<0.0001
Severe AS based on ECHO	ECHO2nd	2.25 (1.95–2.60)	<0.0001
	AVR	219.50 (54.41–885.90)	<0.0001
	AVR OR ECHO2ND	2.31 (1.99–2.68)	<0.0001

Because AVR is rarely coded in the absence of a formal AS diagnosis, few AVR events occurred among undiagnosed patients, inflating these hazard ratios. To investigate this finding, a post hoc analysis was conducted incorporating a 30-day landmark period whereby the patient must be enrolled in the health plan following echocardiogram assessment. Patients were then categorized with and without an AS diagnosis code within the 30 days. Adjusted time-to-AVR analysis for this post hoc analysis demonstrated significantly higher AVR rates among patients receiving timely AS diagnosis compared to those without (moderate AS: HR 16.71, 95% CI 10.78–25.89 [*P* < 0.0001]; severe AS: HR 33.05, 95% CI 19.97–54.70 [*P* < 0.0001]).

## 4 Discussion

In this large, real-world cohort of patients with AS severity identified as moderate or severe through echocardiography, we observed three key findings. First, AS was substantially underdiagnosed, with only 22.0% of moderate and 38.3% of severe AS patients receiving a formal ICD-10 diagnosis within 12 months of echocardiographic detection. Second, significant disparities existed by sex and race, with women and non-White patients experiencing lower diagnosis rates than men and White patients. Third, formal diagnosis was strongly associated with subsequent follow-up care, with diagnosed patients approximately 1.7- to 2.3-fold more likely to receive subsequent imaging or AVR. These findings derive from the Optum Market Clarity database, which enhance generalizability by capturing longitudinal claims and clinical data from a diverse, geographically distributed US population.

The degree of underdiagnosis observed in our study strongly parallels the degree of undertreatment in the US population. Li et al. found that only 48% of patients with severe AS and a guideline indication for AVR received the procedure, with undertreatment persisting despite growth in AVR volumes over an 18-year period [11]. Similarly, Généreux et al. demonstrated that among patients with severe AS, only about 61% received AVR within four years of diagnosis [1].

Several factors likely contribute to the low rates of formal AS diagnosis following echocardiographic detection of moderate or severe AS. First, many echocardiograms are ordered with a particular clinical question in mind, such as assessing ejection fraction during chemotherapy, evaluating syncope or myocardial infarction, or looking for wall motion abnormalities. In such cases, AS may not be the primary focus and therefore could be overlooked. Second, the severity of AS may not be clearly reported despite inclusion of quantitative metrics [5]. The 2025 American Society of Echocardiography reporting guidelines now recommend standardized severity grading and suggest including explicit clinical consultation language when significant AS is identified [12]. Third, AS has an insidious onset, with symptoms that are subtle, gradual, and often attributed to other causes or comorbidities. Interestingly, despite these diagnostic challenges, patients ≥75 years in our cohort had higher diagnosis rates than younger patients (moderate AS: 26.9% vs 18.6%; severe AS: 43.3% vs 34.2%), possibly reflecting greater clinical suspicion for AS in this age group or the perception of AS as a disease of aging. However, even among older patients, the majority remained undiagnosed at 12 months.

Significant disparities by race and sex were evident in our findings. Non-White patients were less likely to receive a formal AS diagnosis, consistent with Crousillat et al.'s findings of lower diagnosis rates among Black and Asian patients [5] and the known undertreatment of racial and ethnic minorities for AVR [13]. These patterns are consistent with broader disparities in echocardiographic utilization; Hyland et al. demonstrated lower TTE utilization intensity among women and racial minorities [14], while Spetko et al. found that populations with greater sociodemographic risk face geographic barriers to imaging access and remain underserved even in urban settings where proximity to imaging centers is not a barrier [15]. Women experienced longer delays to diagnosis, which is particularly concerning given evidence that female patients carry a heavier symptomatic burden in AS [8]. Strange et al. demonstrated that AI-based detection systems can identify unconscious clinical bias in recognizing severe AS, particularly in women, and may help correct the male bias that influences clinical management decisions [16]. Notably, ECI burden was similar between patients who did and did not receive a formal diagnosis, suggesting these disparities are not explained by differences in overall health status.

Our findings suggest that temporal distance from initial detection is an important factor—if AS is not recognized and acted upon early after echocardiographic detection, it may be forgotten or overlooked in subsequent encounters unless something specifically draws attention back to the finding. The strong association we observed between formal diagnosis and likelihood of receiving follow-up care highlights the critical importance of making a formal diagnosis after AS is detected on echocardiography.

Patients with outpatient (versus inpatient) echocardiograms were more likely to receive an AS diagnosis, possibly reflecting differences in clinical context and opportunity for comprehensive evaluation across care settings. Lindman et al. emphasized that quality measurement should start upstream of valve replacement [6]. Systematic approaches such as the AHA Target Aortic Stenosis initiative aim to create automatic alerts and system-wide processes for timely therapy, calling for treatment within 90 days for patients with class I indications. The recently published DETECT AS trial demonstrated that electronic provider notifications (EPNs) significantly improved AVR rates (48.2% vs 37.2%; *P* = 0.009), with the greatest benefit observed in women (OR 2.78), where EPNs helping to lessen sex disparities in treatment rates [17]. EPNs also prolonged survival, with symptomatic patients gaining nearly three weeks of additional life over one year of follow-up [17]. These findings suggest that simple, scalable interventions can improve care quality and reduce demographic disparities in AS management.

Our study has several strengths and limitations. The strengths include the large sample size providing population-level insights into AS diagnosis patterns, the real-world nature of the data, and the use of natural language processing to improve capture of discrete fields beyond claims data alone, enhancing clinical insights and predictive modeling. These approaches are cost-efficient and enable fast analysis across a diverse population.

The surprisingly large degree of missingness in key echocardiographic parameters warrants specific mention. While this constrained some analyses, it also reflects a broader clinical reality: Inconsistent reporting of AS severity metrics on echocardiograms contributes to underdiagnosis and delays in care. This documentation gap is a key focus of the AHA Target AS initiative, which emphasizes the need for standardized, complete reporting of valve findings to facilitate timely recognition and treatment.

Additionally, prior validation studies have demonstrated that ICD codes for AS are highly specific but lack sensitivity, which may contribute to the low rates of formal diagnosis observed in our study [7]. Other limitations include the lack of detailed clinical context for each patient's care decisions. The study period also overlapped with the COVID-19 pandemic, which likely impacted healthcare delivery patterns and patient follow-up.

In conclusion, this study reveals substantial delays and disparities in the formal diagnosis of AS following echocardiographic detection. Given the documented mortality risk of untreated AS and evidence that formal diagnosis is strongly associated with subsequent care, our findings emphasize the need for systematic initiatives to improve recognition and documentation of AS after echocardiographic detection. Programs such as the Target AS initiative and interventions such as EPNs are critical to addressing these care gaps. Future research should focus on developing and implementing standardized protocols for AS diagnosis after echocardiographic detection, particularly for vulnerable populations at risk for underdiagnosis and undertreatment based on race, ethnicity, and sex. Additional investigation into the role of provider specialty, hospital characteristics, echocardiographic study type, and temporal trends on diagnostic patterns may further inform targeted interventions.

## CRediT authorship contribution statement

**Ben Peterson:** Validation, Supervision, Resources, Investigation, Conceptualization, Writing – review & editing. **Jordan B. Strom:** Validation, Supervision, Methodology, Investigation, Conceptualization, Writing – review & editing. **Michael Ryan:** Validation, Software, Resources, Methodology, Investigation, Formal analysis, Data curation, Writing – review & editing. **Candace Gunnarsson:** Validation, Supervision, Project administration, Methodology, Formal analysis, Data curation, Conceptualization, Writing – review & editing, Writing – original draft. **Soumya G. Chikermane:** Supervision, Software, Resources, Project administration, Investigation, Formal analysis, Data curation, Conceptualization, Writing – review & editing. **Seth Clancy:** Supervision, Resources, Project administration, Funding acquisition, Data curation, Conceptualization, Writing – review & editing. **Sammy Elmariah:** Validation, Supervision, Methodology, Investigation, Conceptualization, Writing – review & editing, Writing – original draft.

## Ethical statement

This study was conducted in accordance with the principles of the Declaration of Helsinki. The analysis used fully de-identified, HIPAA-compliant administrative claims data from the Optum® Market Clarity Database (claims + electronic medical records [EMR]) from January 1, 2016, to December 31, 2023. Because the data contain no personally identifiable information and the research did not involve direct interaction with human subjects, the study was determined to be exempt from Institutional Review Board review under 45 CFR 46.104(d)(4). Informed consent was therefore not required.

## Funding sources

This study was supported by 10.13039/100006520Edwards Lifesciences.

## Declaration of competing interest

Dr. Peterson has the following disclosures: research funding from Edwards Lifesciences; consulting/speaking: Egnite inc, Edwards Lifesciences, Medtronic, Amgen, Xenter, CRF. Dr. Strom reports research grants from the National Heart, Lung, and Blood Institute (1R01HL169517, 1R01HL173998), Edwards Lifesciences, EchoIQ, Anumana, Philips Healthcare, Viz.ai, EVERSANA Lifesciences; consulting for Bracco Diagnostics, Edwards Lifesciences, Philips Healthcare, Ultrasight, General Electric Healthcare, and EVERSANA Lifesciences; clinical endpoint committee for Pfizer and Agitated Solutions, and is a member of the scientific advisory boards for Ultromics, HeartSciences, Bristol Myers Squibb, Alnyam, and EchoIQ. Michael Ryan and Candace Gunnarsson are paid consultants to Edwards Lifesciences. Soumya G Chikermane and Seth Clancy are employees of Edwards Lifesciences. Dr Elmariah receives research grants from the National Institutes of Health (R01 HL151838), Patient-Centered Outcomes Research Institute, Edwards Lifesciences, Abbott Vascular, and Medtronic; has received consulting fees from Edwards Lifesciences and LightHearted AI; and holds equity in Prospect Health.

## Figures and Tables

**Table:** 

A.1 Variable	Code	Description
Aortic Stenosis	I06.0	Rheumatic aortic stenosis
Aortic Stenosis	I06.2	Rheumatic aortic stenosis with insufficiency
Aortic Stenosis	I35.0	Nonrheumatic aortic (valve) stenosis
Aortic Stenosis	I35.2	Nonrheumatic aortic (valve) stenosis with insufficiency
AVR	02RF07Z	Replacement of Aortic Valve with Autologous Tissue Substitute, Open Approach
AVR	02RF08Z	Replacement of Aortic Valve with Zooplastic Tissue, Open Approach
AVR	02RF0JZ	Replacement of Aortic Valve with Synthetic Substitute, Open Approach
AVR	02RF0KZ	Replacement of Aortic Valve with Nonautologous Tissue Substitute, Open Approach
AVR	02RF37Z	Replacement of Aortic Valve with Autologous Tissue Substitute, Percutaneous Approach
AVR	02RF38Z	Replacement of Aortic Valve with Zooplastic Tissue, Percutaneous Approach
AVR	02RF3JZ	Replacement of Aortic Valve with Synthetic Substitute, Percutaneous Approach
AVR	02RF3KZ	Replacement of Aortic Valve with Nonautologous Tissue Substitute, Percutaneous Approach
AVR	02RF47Z	Replacement of Aortic Valve with Autologous Tissue Substitute, Percutaneous Endoscopic Approach
AVR	02RF48Z	Replacement of Aortic Valve with Zooplastic Tissue, Percutaneous Endoscopic Approach
AVR	02RF4JZ	Replacement of Aortic Valve with Synthetic Substitute, Percutaneous Endoscopic Approach
AVR	02RF4KZ	Replacement of Aortic Valve with Nonautologous Tissue Substitute, Percutaneous Endoscopic Approach
AVR	02RF37H	Replacement of Aortic Valve with Autologous Tissue Substitute, Transapical, Percutaneous Approach
AVR	02RF38H	Replacement of Aortic Valve with Zooplastic Tissue, Transapical, Percutaneous Approach
AVR	02RF3JH	Replacement of Aortic Valve with Synthetic Substitute, Transapical, Percutaneous Approach
AVR	02RF3KH	Replacement of Aortic Valve with Nonautologous Tissue Substitute, Transapical, Percutaneous Approach
ECHO	B244YZZ	Ultrasonography of Right Heart using Other Contrast
ECHO	B244ZZ3	Ultrasonography of Right Heart, Intravascular
ECHO	B244ZZ4	Ultrasonography of Right Heart, Transesophageal
ECHO	B244ZZZ	Ultrasonography of Right Heart
ECHO	B245YZZ	Ultrasonography of Left Heart using Other Contrast
ECHO	B245ZZ3	Ultrasonography of Left Heart, Intravascular
ECHO	B245ZZ4	Ultrasonography of Left Heart, Transesophageal
ECHO	B245ZZZ	Ultrasonography of Left Heart
ECHO	B246YZZ	Ultrasonography of Right and Left Heart using Other Contrast
ECHO	B246ZZ3	Ultrasonography of Right and Left Heart, Intravascular
ECHO	B246ZZ4	Ultrasonography of Right and Left Heart, Transesophageal
ECHO	B246ZZZ	Ultrasonography of Right and Left Heart
ECHO	B24BYZZ	Ultrasonography of Heart with Aorta using Other Contrast
ECHO	B24BZZ3	Ultrasonography of Heart with Aorta, Intravascular
ECHO	B24BZZ4	Ultrasonography of Heart with Aorta, Transesophageal
ECHO	B24BZZZ	Ultrasonography of Heart with Aorta
ECHO	B24DYZZ	Ultrasonography of Pediatric Heart using Other Contrast
ECHO	B24DZZ4	Ultrasonography of Pediatric Heart, Transesophageal
ECHO	B24DZZZ	Ultrasonography of Pediatric Heart
ECHO	93303	Transthoracic echocardiography for congenital cardiac anomalies; complete
ECHO	93304	Transthoracic echocardiography for congenital cardiac anomalies; follow-up or limited study
ECHO	93306	Echocardiography, transthoracic, real-time with image documentation (2D), includes M-mode recording, when performed, complete, with spectral Doppler echocardiography, and with color flow Doppler echocardiography
ECHO	93307	Echocardiography, transthoracic, real-time with image documentation (2D), includes M-mode recording, when performed, complete, without spectral or color Doppler echocardiography
ECHO	93308	Echocardiography, transthoracic, real-time with image documentation (2D), includes M-mode recording, when performed, follow-up or limited study
ECHO	93312	Echocardiography, transesophageal, real-time with image documentation (2D) (with or without M-mode recording); including probe placement, image acquisition, interpretation and report
ECHO	93313	Echocardiography, transesophageal, real-time with image documentation (2D) (with or without M-mode recording); placement of transesophageal probe only
ECHO	93314	Echocardiography, transesophageal, real-time with image documentation (2D) (with or without M-mode recording); image acquisition, interpretation and report only
ECHO	93315	Transesophageal echocardiography for congenital cardiac anomalies; including probe placement, image acquisition, interpretation and report
ECHO	93316	Transesophageal echocardiography for congenital cardiac anomalies; placement of transesophageal probe only
ECHO	93317	Transesophageal echocardiography for congenital cardiac anomalies; image acquisition, interpretation and report only
ECHO	93318	Echocardiography, transesophageal (TEE) for monitoring purposes, including probe placement, real time 2-dimensional image acquisition and interpretation leading to ongoing (continuous) assessment of (dynamically changing) cardiac pumping function and to therapeutic measures on an immediate time basis
ECHO	93350	Echocardiography, transthoracic, real-time with image documentation (2D), includes M-mode recording, when performed, during rest and cardiovascular stress test using treadmill, bicycle exercise and/or pharmacologically induced stress, with interpretation and report;
ECHO	93351	Echocardiography, transthoracic, real-time with image documentation (2D), includes M-mode recording, when performed, during rest and cardiovascular stress test using treadmill, bicycle exercise and/or pharmacologically induced stress, with interpretation and report; including performance of continuous electrocardiographic monitoring, with supervision by a physician or other qualified health care professional
ECHO	93355	Echocardiography, transesophageal (TEE) for guidance of a transcatheter intracardiac or great vessel(s) structural intervention(s) (e.g., TAVR, transcatheter pulmonary valve replacement, mitral valve repair, paravalvular regurgitation repair, left atrial appendage occlusion/closure, ventricular septal defect closure) (peri-and intra-procedural), real-time image acquisition and documentation, guidance with quantitative measurements, probe manipulation, interpretation, and report, including diagnostic transesophageal echocardiography and, when performed, administration of ultrasound contrast, Doppler, color flow, and 3D
ECHO	93662	Intracardiac echocardiography during therapeutic/diagnostic intervention, including imaging supervision and interpretation (List separately in addition to code for primary procedure)

**Table:** 

Variable	Moderate AS		Severe AS	
	Hazard ratio 95% CI	*P* value	Hazard ratio 95% CI	P value
Time to second ECHO				
Time to AS diagnosis *Time varying covariate*	1.68 (1.50–1.89)	<0.0001	2.25 (1.95–2.60)	<0.0001
Age at first ECHO	1.00 (0.99–1.00)	0.1954	1.00 (0.99–1.00)	0.5083
Female	0.77 (0.70–0.86)	<0.0001	0.78 (0.68–0.89)	0.0003
Asian	0.89 (0.47–1.67)	0.7109	0.75 (0.33–1.69)	0.486
Black	0.89 (0.64–1.22)	0.4567	0.80 (0.50–1.28)	0.3533
Caucasian	0.79 (0.59–1.06)	0.121	0.81 (0.53–1.24)	0.3387
Midwest	0.90 (0.62–1.32)	0.5945	0.76 (0.50–1.15)	0.1967
Northeast	1.39 (0.93–2.08)	0.1093	0.90 (0.57–1.42)	0.6448
South	0.96 (0.66–1.39)	0.8203	0.82 (0.53–1.26)	0.3635
West	0.92 (0.62–1.36)	0.6669	0.89 (0.55–1.43)	0.6336
Elixhauser Comorbid Score	1.05 (1.03–1.07)	<0.0001	1.02 (0.99–1.04)	0.2075

Time to AVR				
Time to AS diagnosis *Time varying covariate*	102.3 (37.12–281.7)	<0.0001	219.5 (54.41–885.9)	<0.0001
Age at first ECHO	0.97 (0.96–0.99)	0.0002	0.98 (0.97–0.98)	<0.0001
Female	0.42 (0.28–0.63)	<0.0001	0.50 (0.38–0.66)	<0.0001
Asian	0.00 (0.00 - .)	0.9789	0.00 (0.00 - 9E303)	0.9796
Black	0.09 (0.03–0.31)	0.0001	0.36 (0.15–0.87)	0.0234
Caucasian	0.28 (0.13–0.62)	0.0017	0.65 (0.35–1.22)	0.1795
Midwest	0.96 (0.29–3.13)	0.942	0.82 (0.38–1.76)	0.6096
Northeast	1.66 (0.45–6.04)	0.4444	1.41 (0.63–3.13)	0.4042
South	1.39 (0.43–4.52)	0.5821	1.13 (0.52–2.48)	0.7589
West	0.74 (0.21–2.66)	0.6473	0.95 (0.40–2.23)	0.8983
Elixhauser Comorbid Score	0.91 (0.85–0.97)	0.0046	0.93 (0.88–0.98)	0.0056

Time to 2nd ECHO or AVR (composite)				
Time to AS diagnosis *Time varying covariate*	1.67 (1.48–1.87)	<0.0001	2.31 (1.99–2.68)	<0.0001
Age at first ECHO	1.00 (0.99–1.00)	0.2967	1.00 (0.99–1.00)	0.5295
Female	0.77 (0.70–0.86)	<0.0001	0.75 (0.65–0.87)	<0.0001
Asian	0.92 (0.48–1.74)	0.7919	0.82 (0.36–1.88)	0.6377
Black	0.92 (0.66–1.27)	0.5961	0.89 (0.54–1.47)	0.6447
Caucasian	0.81 (0.60–1.10)	0.1792	0.91 (0.58–1.44)	0.6912
Midwest	0.92 (0.62–1.35)	0.6595	0.79 (0.51–1.24)	0.3077
Northeast	1.41 (0.93–2.12)	0.1031	0.98 (0.61–1.59)	0.9472
South	0.98 (0.67–1.43)	0.9265	0.86 (0.55–1.35)	0.5089
West	0.95 (0.64–1.42)	0.8056	0.86 (0.52–1.42)	0.5601
Elixhauser Comorbid Score	1.05 (1.03–1.07)	<0.0001	1.02 (0.99–1.04)	0.2201
